# Liquid Crystal Orientation and Shape Optimization for the Active Response of Liquid Crystal Elastomers

**DOI:** 10.3390/polym16101425

**Published:** 2024-05-17

**Authors:** Jorge Luis Barrera, Caitlyn Cook, Elaine Lee, Kenneth Swartz, Daniel Tortorelli

**Affiliations:** Lawrence Livermore National Laboratory, 7000 East Ave, Livermore, CA 94550, USA; krikorian3@llnl.gov (C.C.); lee1040@llnl.gov (E.L.); swartz10@llnl.gov (K.S.); tortorelli2@llnl.gov (D.T.)

**Keywords:** liquid crystal elastomer, design optimization, finite element analysis, active material, responsive material, smart material, soft actuation

## Abstract

Liquid crystal elastomers (LCEs) are responsive materials that can undergo large reversible deformations upon exposure to external stimuli, such as electrical and thermal fields. Controlling the alignment of their liquid crystals mesogens to achieve desired shape changes unlocks a new design paradigm that is unavailable when using traditional materials. While experimental measurements can provide valuable insights into their behavior, computational analysis is essential to exploit their full potential. Accurate simulation is not, however, the end goal; rather, it is the means to achieve their optimal design. Such design optimization problems are best solved with algorithms that require gradients, i.e., sensitivities, of the cost and constraint functions with respect to the design parameters, to efficiently traverse the design space. In this work, a nonlinear LCE model and adjoint sensitivity analysis are implemented in a scalable and flexible finite element-based open source framework and integrated into a gradient-based design optimization tool. To display the versatility of the computational framework, LCE design problems that optimize both the material, i.e., liquid crystal orientation, and structural shape to reach a target actuated shapes or maximize energy absorption are solved. Multiple parameterizations, customized to address fabrication limitations, are investigated in both 2D and 3D. The case studies are followed by a discussion on the simulation and design optimization hurdles, as well as potential avenues for improving the robustness of similar computational frameworks for applications of interest.

## 1. Introduction

Liquid crystal elastomers (LCEs) are a class of stimuli-responsive polymers that can perform large reversible deformations due to environmental changes (e.g., temperature) or local stimuli (e.g., focused UV/IR light) by programming the orientation of the liquid crystals [1]. This behavior has benefited several application domains, such as soft actuators [2], directional leaping [1,3], and origami design [4], among others. Optimizing the placement and orientation of the liquid crystals (LCs) mesogens within a structure can open the door to additional innovative functionalities [5,6,7]. Moreover, these optimized structures can be fabricated via surface patterning or additive manufacturing, facilitating the exploration of complex geometries and, consequently, expanding the design space. This work leverages such design freedoms and introduces a computational approach to systematically optimize the liquid crystal placement and orientation, and the structure shape to achieve remarkable performances.

The systematic design optimization with responsive materials, often referred to as active or smart materials, sits at the forefront of both material science and engineering. A broad spectrum of design tools including data-driven artificial intelligence (AI) techniques and gradient-based optimization have been developed for this purpose [8,9,10,11]. However, AI methods require abundant data, which is lacking for these responsive structures. Moreover, the existing AI literature related to this field focuses primarily on structural mechanics [12,13,14]. On the other hand, gradient-based optimization approaches have been successfully applied to design responsive structures comprised of, for example, shape memory polymers [15,16], piezoelectric materials [10,17], and LCEs [18,19].

The cornerstone of gradient-based design optimization is sensitivity analysis (SA), i.e., the computation of the derivatives of the optimization cost and constraint functions with respect to its material, geometric, and load parameters [20,21,22,23]. These gradients also play a pivotal role in, for example, perturbation analysis, mesh adaptivity, and uncertainty quantification, to name a few [24,25,26]. Despite their extensive benefit, SA is rarely performed due to cumbersome formulations, especially for the nonlinear response that characterizes LCE structures; see [27,28,29]. Fortunately, these challenges can be circumvented via automatic differentiation (AD) tools [30,31]. Notably, the time and effort required to implement AD has been significantly reduced in the past decade, and nowadays, an extensive catalog of open source AD libraries for SA is readily available [32,33,34,35].

Multiple strategies have been proposed to effectively simulate the stimuli response, i.e., motion, of active structures [36,37,38,39]. It is widely recognized that these simulations involve the solution of coupled nonlinear partial differential equations (PDEs), which require specialized strategies to address both numerical and physical instabilities. LCEs are no different in this regard, as stimuli response predictions must capture the behavior of the LC mesogens in the material’s architecture (i.e., the LCE anisotropy) [4,40,41,42,43]. To model this phenomena, LCE continuum material models use a scalar- or tensor-order parameter to quantify the alignment of the LCE mesogens. Such models have been validated for both small- and large-strain deformations resulting from a variety of loadings, e.g., thermal and light [44,45,46,47].

In this work, we adopt the hyperelastic material model described in [48] to model the response of LCE materials. We leverage an in-house next generation finite element analysis code, i.e., Serac, to facilitate the material model implementation effort and benefit from its high-performance computing (HPC) and AD capabilities. This stands in stark contrast to commercial analysis software alternatives, where considerable effort would be needed to accommodate new physics models, let alone the SA [49,50,51,52,53].

To the best of the authors’ knowledge, neither shape optimization nor combined shape and LC orientation optimization have been investigated for LCEs. Related work relies on simplified material models that may not adequately capture the intricate response exhibited by LCE materials [18,19], or explores only two-dimensional LC orientation optimization [47]. Thus, the primary contributions of this manuscript are the combined LC orientation and shape optimization of LCE structures in a computational framework that is amenable to multiple printing technologies (e.g., direct ink write and digital light processing) employed. Design problems for relevant applications are explored, including achieving target actuated shapes and maximizing energy absorption. Additionally, we provide a summary of the current challenges encountered and future work needed to unleash the full potential of LCE structures.

The remainder of this manuscript is organized as follows. Section 2 describes the nonlinear finite strain LCE model and the finite element analysis for the quasi-static boundary value problem. The formulations for the LC orientation and shape sensitivity analyses are summarized in Section 3. The solved orientation and shape optimization problems of Section 4 demonstrate the versatility of the proposed framework to design 2D and 3D LCE structures. Section 5 provides remarks regarding current challenges and directions for future work. Finally, a summary is provided in Section 6.

## 2. Problem Description

**Figure 1:**
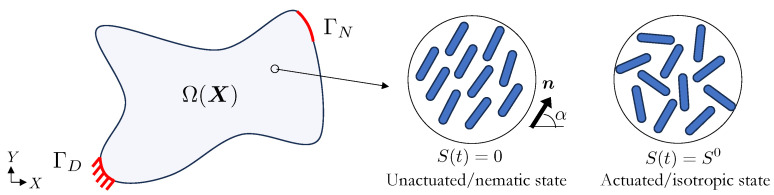
Design domain with boundary conditions (**left**) and a zoomed in region that shows the orientation of the LC in the unactuated/nematic (**center**) and actuated/isotropic (**right**) states.

We consider a body B in the undeformed configuration $\Omega \subset R^{n_{d}}$ for $n_{d}=\left[2,3\right]$ spatial dimensions with external boundary $\Gamma \subset R^{d-1}$. Complementary boundaries of Γ that are subjected to prescribed Dirichlet (essential) and Neumann (natural) boundary conditions are denoted by $\Gamma_{D}$ and $\Gamma_{N}$, respectively, as seen in Figure 1.

Without loss of generality, we adopt the experimentally validated LCE material model used in [48]. The strain energy density for the LCE hyperelastic material is

(1) $$\begin{array}{c} \psi \left(F\right)=\frac{\lambda }{2}{\left(tr\left(E\right)\right)}^{2}+\mu \left(E\cdot E\right)-\frac{1}{2}\beta \left(S^{0}-S\left(t\right)\right)\left(3n^{T}n-I\right)\cdot E \end{array}$$

where λ and μ are the first and second Lamé coefficients. These coefficients can be calculated from Young’s modulus E and the Poisson ratio ν via $\lambda =\frac{E\nu }{\left(1+\nu )(1-2\nu \right)}$ and $\mu =\frac{E}{2(1+\nu )}$, respectively [54]. The Green–Lagrange strain tensor $E=\frac{1}{2}\left(FF^{⊤}-I\right)$ is a function of the deformation gradient $F=I+\frac{\partial u}{\partial X}$, where I is the identity tensor, u is the displacement vector, and X∈Ω is the location of a material particle in the undeformed configuration. The first and second terms on the right-hand side of Equation (1) model an isotropic Saint Venant material, while the third term models the anisotropic nematic order effect. The parameter β defines the degree of anisotropy, while $S^{0}$ and S(t) define the initial and instantaneous scalar-order parameters, and *t* is the pseudo time as described in Section 2.3. The unit vector n indicates the alignment of the LCs embedded in the elastomer, i.e., the LC orientation, cf. Figure 1.

The initial order parameter $S^{0}\geq 0$ indicates the highest possible level of ordering, while $S\left(t\right)\in \left[0,S^{0}\right]$ models the transition from the unactuated/nematic state S(t)=0 to the actuated/isotropic state $S\left(t\right)=S^{0}$, cf. Figure 1. Normally, *S* is controlled by an external actuation mechanism, e.g., thermal, cf. [36,46]. Without loss of generality, we have omitted such dependence, i.e., *S* evolves by a prescribed update strategy.

Under our hyperelastic material assumption, the First Piola stress is defined as

(2) $$\begin{array}{c} P=F\frac{\partial \psi }{\partial E}=F\left[\lambda tr\left(E\right)I+2\mu E-\frac{1}{2}\beta \left(S^{0}-S\left(t\right)\right)\left(3n^{T}n-I\right)\right], \end{array}$$

and the symmetric Cauchy stress σ as

(3) $$\begin{array}{c} \sigma =J^{-1}PF^{T}, \end{array}$$

where J=det(F).

In our examples, we consider two stress-free configurations. Our interpretation of the energy ψ differs from [48]. It is our view that the stress-free LCE structure is pre-strained when manufactured and, as a response to a stimulus, the material transitions to the strain-free state when reaching the isotropic configuration. Hence, the isotropic, i.e., the actuated, configuration should be used as the underformed reference configuration instead of the nematic, i.e., unactuated, configuration. In the fully nematic configuration, S(t)=0, and in the fully isotropic configuration, $S\left(t\right)=S^{0}$. In the latter, zero stress, i.e., P=0, implies E=0. In the former, zero stress implies that we have a nonzero strain $E=\frac{\beta S^{0}}{2\mu }\left(3n^{T}n-I\right)$. Notably, as the stress-free nematic configuration transitions to the stress-free isotropic configuration, it axially contracts $-\frac{\beta S^{0}}{\mu }$ in the n direction, and expands $\frac{\beta S^{0}}{2\mu }$ in the directions perpendicular to n. It neither shears nor changes volume.

### 2.1. Design Parameterization

In the optimization problems considered here, the LCE structure design is parameterized by a finite element mesh over the domain Ω via the $n_{p}$ design parameters $p={\left[p_{1},p_{2},\cdots ,p_{n_{p}}\right]}^{⊤}$ that describe the orientation of the LC mesogens n and/or the shape via perturbations of the mesh node coordinates ΔX. The layer-by-layer additive manufacturing of the LCE structure is such that the mesogen orientation in each element is restricted to the X−Y principal plane. This simplification means that the orientation is defined by the element inclination angle α, i.e., n=(cos(α),sin(α),0), cf. Figure 1. Furthermore, the shape optimization method parameterizes the topology, preserving shape changes as described in [55]. Note that despite this manuscript focusing on LC phases, the design parameterization can be formulated such that it appropriately simulates cholesteric (i.e., chiral nematic) or blue (double twist cylinders packed in cubic lattices) phases.

To formulate a well-posed optimization problem, the design fields are filtered to prevent highly oscillatory orientations and complex shapes [55,56]. Thus, we optimize the parameters p, but we model the structure using the filtered parameters $\tilde{p}$ that define the filtered orientation $\tilde{n}$ and shape perturbations $\tilde{\Delta X}$.

### 2.2. Filter Analysis

We implement an energy filter which defines the filtered design field $\tilde{p}$ as the solution of a PDE defined on Ω [57]. The weak form of this governing equation seeks $\tilde{p}\in W$ such that

(4) $$R^{EF}\left(\tilde{p}\right)=\int_{\Omega }\left(\nabla v\cdot r^{2}\frac{\partial \zeta (\nabla \tilde{p})}{\partial \nabla \tilde{p}}+v\cdot \left(\tilde{p}-p\right)\right)d\Omega ,=0.$$

where the parameter *r* implicitly defines the smoothness of $\tilde{p}$ relative to p, with larger values providing more smoothness. The “energy” ζ is defined differently for LC orientation and shape parameters. For LC orientation, $\zeta =\nabla \tilde{\alpha }\cdot \nabla \tilde{\alpha }$, and for the shape perturbations, $\zeta =tr\left(\nabla \tilde{\Delta X}\right)/(det{\left(\nabla \tilde{\Delta X)}\right)}^{1/n_{d}}$. For LC orientation parameters, $v\in W=H^{1}$, while for shape parameters

(5) $$v\in W=\{\tilde{p}\in H^{1}\left(\Omega \right):\tilde{p}\left(X\right)=\tilde{\Delta X}\left(X\right)=0\;for\;X\in \Gamma_{D}^{\Delta X}\}.$$

Here, $\Gamma_{D}^{\Delta X}$ represents the surface where the Dirichlet conditions are applied for the shape parameters. For shape optimization, $\tilde{\Delta X}$ is interpolated from the nodal values via the usual Lagrangian finite element basis functions [55]. For LC orientation, α and $\tilde{\alpha }$ are parameterized to be piecewise uniform over the elements, i.e., $\alpha ,\tilde{\alpha }\in L^{2}$. However, W requires α and $\tilde{\alpha }$ to be smooth to compute their spatial gradients, i.e., $\alpha ,\tilde{\alpha }\in H^{1}$. To resolve this inconsistency, α is projected to $H^{1}$ to solve Equation (4) for the filtered design field $\tilde{\alpha }\in H^{1}$, which is subsequently projected back to $L^{2}$.

### 2.3. LCE Forward Analysis

In the LCE simulations, we evaluate the displacement u∈H that solves

(6) $$R^{LCE}\left(u\right)=\int_{\Omega }\nabla w:P\left(\nabla u\right)\;d\Omega =0,$$

for all w∈H, where

(7) $$H=\{u\in H^{1}\left(\Omega \right):u\left(X\right)=0\;for\;X\in \Gamma_{D}\}.$$

Without loss of generality, we assume homogeneous Dirichlet boundary conditions on $\Gamma_{D}$ and null applied body and surface traction. Note that the boundary regions where the Dirichlet conditions are applied for the filter and LCE analyses generally differ. The material response is obtained as the order parameter S(t) linearly decreases such that $S\left(t\right)=S^{0}\left(1-\frac{t}{t_{max}}\right)$, where $t_{max}$ is the total simulation time, i.e., the pseudo time $t\in [0,t_{max}]$.

The nonlinear Equation (6) is solved using the iterative Newton–Raphson method. In this method, if the residual of the current solution guess u is not converged, i.e., if $|R^{LCE}\left(u\right)|\geq \epsilon_{R}$ for a small tolerance $\epsilon_{R}$, then the next guess becomes u=u+Δu, where Δu solves the linear equation

(8) $$\frac{\partial R^{LCE}\left(u\right)}{\partial u}\Delta u=-R^{LCE}\left(u\right)$$

in which $\frac{\partial R^{LCE}}{\partial u}$ is the tangent stiffness matrix that is computed via AD. This procedure is performed for all pseudo time steps. Sufficiently small time steps are used such that the Newton algorithm converges in just a few iterations.

## 3. Optimization Framework

Upon solving Equation (6) to the terminal time $t_{max}$, we can evaluate any number of the performance metrics (e.g., total energy absorbed, maximum stress) via the response functional

(9) $$F=G\left(u,\tilde{p}\right)=\int_{\Omega }\Pi \left(u,\nabla u,\tilde{p}\right)d\Omega ,$$

where Π is the general integrand function. Such metrics are used to define the optimization cost and constraint functions.

**Figure 2:**
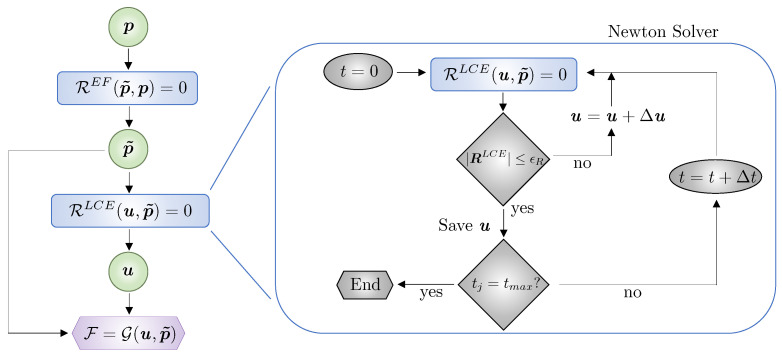
Graph for the forward analysis including the Newton solver block to compute the LCE structure’s cost and constraint functions.

A graph that describes the forward analysis needed to compute the filtered design parameters, displacement, and performance metrics is illustrated in Figure 2. The block on the right encapsulates the transient Newton solver. The design parameters p are used to solve the filter equation $R^{EF}\left(\tilde{p},p\right)=0$ and obtain their smoothed versions $\tilde{p}$, which are then used to define the LC orientation and/or geometry of the domain in the LCE analysis $R^{LCE}\left(u,p\right)=0$ to compute u. Subsequently, we calculate the cost and constraint functions $F=G(u,\tilde{p})$.

**Figure 3:**
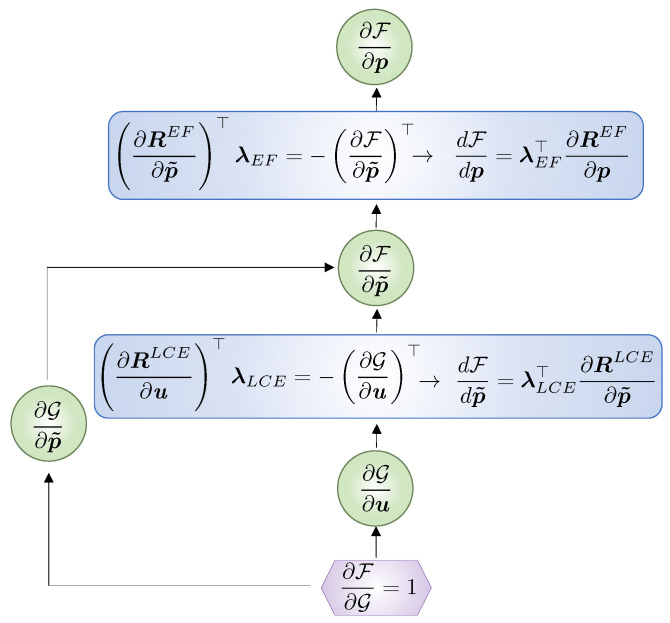
Graph for the adjoint sensitivity analysis.

We traverse the graph in Figure 2 backwards to compute the gradients needed for optimization via the adjoint method, cf. Figure 3. The adjoint method is used due to its computational efficiency for the design problems considered here since the number of response functions is far less than the number of design parameters; for details, the reader is referred to [21,22,58].

In the optimization problem derivations, we reintroduce the cost or constraint response function as

(10) $$F=G(u\left(\tilde{p}\right),\tilde{p}),$$

to denote the displacement’s u dependencies on the filtered design, $\tilde{p}$. We derive the sensitivities with respect to the filtered design parameters

(11) $$\frac{dF}{d{\tilde{p}}_{i}}=\frac{\partial G}{\partial {\tilde{p}}_{i}}+\frac{\partial G}{\partial u}\frac{\partial u}{\partial {\tilde{p}}_{i}}.$$

To resolve the unknown derivative $\frac{\partial u}{\partial {\tilde{p}}_{i}}$, we include all dependencies and express Equation (6) as

(12) $$R^{LCE}\left(u\left(\tilde{p}\right),\tilde{p}\right)=0.$$

Differentiating Equation (12), we obtain

(13) $$\frac{\partial R^{LCE}}{\partial p_{i}}+\frac{\partial R^{LCE}}{\partial \tilde{p}}\frac{\partial \tilde{p}}{\partial p_{i}}=0.$$

Multiplying this by the adjoint vector $\lambda_{LCE}$, adding it to Equation (11) and rearranging yields

(14) $$\begin{array}{cc} \frac{dF}{dp_{i}}\; & =\left(\frac{\partial G}{\partial u}+{\lambda_{LCE}}^{⊤}\frac{\partial R^{LCE}}{\partial u}\right)\frac{\partial u}{\partial {\tilde{p}}_{i}}+\frac{\partial G}{\partial {\tilde{p}}_{i}}+{\lambda_{LCE}}^{⊤}\frac{\partial R^{LCE}}{\partial {\tilde{p}}_{i}}. \end{array}$$

The unknown derivative $\frac{\partial u}{\partial {\tilde{p}}_{i}}$ is annihilated from the above by solving the following adjoint problem for the heretofore arbitrary $\lambda_{LCE}$:

(15) $${\left(\frac{\partial R^{LCE}}{\partial u}\right)}^{⊤}\lambda_{LCE}=-{\left(\frac{\partial G}{\partial u}\right)}^{⊤}\;.$$

As such, the sensitivity (Equation (11)) reduces to

(16) $$\frac{dF}{d{\tilde{p}}_{i}}=\frac{\partial G}{\partial {\tilde{p}}_{i}}+\lambda_{LCE}^{⊤}\frac{\partial R^{LCE}}{\partial {\tilde{p}}_{i}}\;.$$

Note that the $\frac{\partial }{\partial \tilde{p}}$ derivatives imply either LC orientation (i.e., $\frac{\partial G}{\partial n}$) or shape (i.e., $\frac{\partial G}{\partial X}$) derivatives. Material (i.e., LC orientation) derivatives are relatively easy to compute as opposed to shape derivatives. However, this difference is moot, as we compute the derivatives $\frac{\partial G}{\partial u}$, $\frac{\partial G}{\partial \tilde{p}}$, and $\frac{\partial R^{LCE}}{\partial \tilde{p}}$ using AD.

Next, we derive the sensitivities with respect to the unfiltered design field p, i.e.,

(17) dFdpi=∂F∂pi0+∂F∂p˜∂p˜∂pi.

The explicit dependency $\frac{\partial F}{\partial p_{i}}$ is eliminated by construction since the performance metrics are only described by $\tilde{p}$. Following the above steps, we express the filter residual in Equation (4) as

(18) $$R^{EF}\left(\tilde{p}\left(p\right),p\right)=0,$$

to denote the filtered design’s $\tilde{p}$ dependence on the parameters p. Differentiating the filter Equation (18) reveals

(19) $$\frac{\partial R^{EF}}{\partial p_{i}}+\frac{\partial R^{EF}}{\partial \tilde{p}}\frac{\partial \tilde{p}}{\partial p_{i}}=0.$$

Multiplying this by the adjoint vector $\lambda_{EF}$, adding it to Equation (17) and rearranging results in

(20) $$\frac{dF}{dp_{i}}=\left(\frac{\partial F}{\partial \tilde{p}}+\lambda_{EF}^{⊤}\frac{\partial R^{EF}}{\partial \tilde{p}}\right)\frac{\partial \tilde{p}}{\partial p_{i}}+\lambda_{EF}^{⊤}\frac{\partial R^{EF}}{\partial p_{i}}.$$

In this case, the unknown derivative $\frac{\partial \tilde{p}}{\partial p_{i}}$ is annihilated from the above by solving a second adjoint problem for the hereto arbitrary $\lambda_{EF}$

(21) $${\left(\frac{\partial R^{EF}}{\partial p_{i}}\right)}^{⊤}\lambda_{EF}=-{\left(\frac{\partial F}{\partial {\tilde{p}}_{i}}\right)}^{⊤},$$

whereupon the design sensitivity reads

(22) $$\frac{dF}{dp_{i}}=\lambda_{EF}^{⊤}\frac{\partial R^{EF}}{\partial p_{i}}.$$

Here, the derivatives $\frac{\partial F}{\partial \tilde{p}}$ and $\frac{\partial R^{EF}}{\partial p}$ are computed via AD.

## 4. Case Studies

We now optimize LC orientation and shape to design LCE structures to achieve the desired deformations and maximal energy absorption, and showcase the proposed methodology’s versatility and robustness across applications. For the sake of consistency with the literature, we henceforth refer to the isotropic state as the actuated configuration, and the nematic state as the unactuated configuration.

The initial designs and their applied boundary conditions for all cases are specified below. Relevant material and analysis parameters for all examples are noted in Table 1. These material properties and parameters were derived from experimental data.

The forward and adjoint analyses are solved by the 3D implicit nonlinear multi-physics finite element code Serac ([35]) that heavily leverages a C++ library for Modular Finite Element Methods, MFEM (Version 4.6 [34]). Conformal 2D meshes are composed of either four-node quadrilateral or three-node triangular elements, and 3D meshes of eight-node hexahedra elements. We interpolate the unknowns at any material point X using Lagrangian interpolation functions. The direct linear solver Strumpack [59] is used to solve the Newton update and adjoint problems. Serac exploits AD to compute the necessary derivatives mentioned above. Hence, our lean implementation requires only defining the stress tensor in Equation (3), and the integrands in Equations (4), (6) and (9).

The orientation field α is piecewise uniform over the finite elements and the element angle orientations $\alpha_{i}$ are the design parameters subject to the limits $\alpha_{min}=-90^{\circ }$ and $\alpha_{max}=90^{\circ }$. This design field is filtered according to Equation (4) using only homogeneous Neumann boundary conditions to obtain $\tilde{\alpha }$, which is also piecewise uniform over the mesh (after performing the appropriate projections discussed above). Hence, each element has its own orientation ${\tilde{\alpha }}_{i}$. Similarly, the shape parameters ΔX are filtered by solving Equation (4) with its corresponding energy function to obtain $\tilde{\Delta X}$. Lower and upper limits $\Delta X_{min}$ and $\Delta X_{max}$ for the shape parameters ΔX and the boundary $\Gamma_{D}^{\Delta X}$ (see Equation (5)) are provided below per case study.

The nonlinear programming Method of Moving Asymptotes (MMA, [60]) algorithm is used to solve the optimization problems. In the optimization, convergence is achieved when the change in value of the cost function for three consecutive iterations is less than $1\times 10^{-3}$. The Livermore Design Optimization code LiDO (Version 0.2.0, [61]) is used to define the design parameterizations, access the optimization solvers, and automatically traverse the graphs of Figure 2 and Figure 3.

### 4.1. Lc Orientation Optimization of Soft Gripper

**Figure 4:**
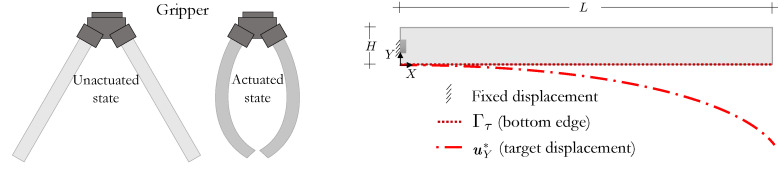
Soft gripper LC orientation design: (**left**) LCE gripper in both the initially unactuated (nematic) and actuated (isotropic) states; and (**right**) design domain and desired deformation.

In this first example, we optimize the LC orientation of an LCE soft gripper to obtain a desired shape change when actuated, cf. the unactuated and actuated configurations in Figure 4. Using domain symmetry, we assign Ω to the rectangular unactuated configuration of size L=50 [mm] by H=5 [mm], which is clamped over the middle fifth of the left edge. The order parameter is then decreased to zero to obtain the target actuated configuration such that the displacement of the bottom edge is $u_{Y}^{*}\left(X\right)=-0.50\;X_{1}^{2}/L\cdot e_{2}$.

To obtain this target displacement, we minimize the root mean square discrepancy between the actuated LCE gripper displacement u along the bottom edge $\Gamma_{\tau }$ and the target displacement $u_{Y}^{*}$ over $\Gamma_{\tau }$, i.e.,

(23) $$\begin{array}{cc} \min_{\alpha }\;\;\;\;\;\;\tau & =\int_{\Gamma_{\tau }}{\left|u-u_{Y}^{*}\right|}^{2}d\Gamma \\ \alpha_{1},\alpha_{2},\ldots ,\alpha_{n_{elems}} & \in [\alpha_{min},\alpha_{max}], \end{array}$$

**Figure 5:**
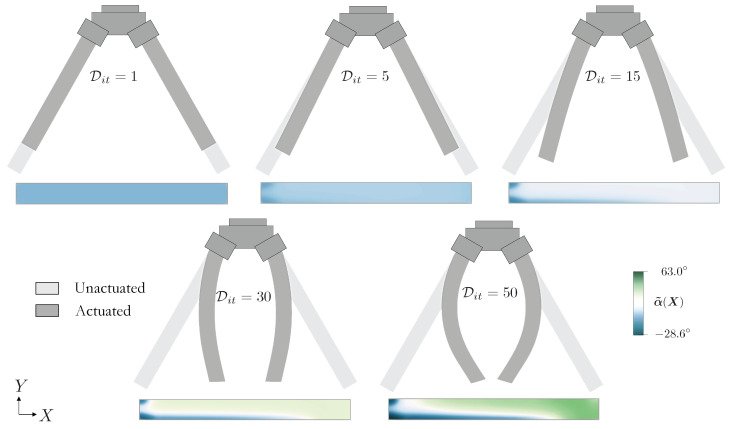
Designs corresponding to optimization iterates $D_{it}=\left[1,5,15,30,50\right]$ showing LC orientation (colored horizontal strip), unactuated (light grey) and actuated (dark gray) configurations.

Figure 5 shows the evolution of the design, i.e., LC orientation and their unactuated and actuated configurations at optimization iterations $D_{it}=\left[1,5,15,30,50\right]$. As the optimization progresses, the orientation of the LCs changes from an uniform constant field of α=0.0 to a spatially varying field characterized by a positive angle over most of the upper region, which is approximately reflected over the lower region. Clearly, the optimization produces the desired shape. The optimization converges after 50 iterations, and the discrepancy τ reduces from its initial normalized value of 1.0 to 0.0015.

### 4.2. Shape Optimization of a Leaping LCE Strip

**Figure 6:**
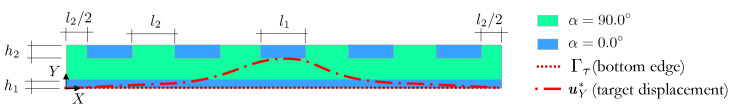
Initial design of leaping structure with two distinct LC orientation regions and its desired deformation.

In the second problem, we design a leaping structure via shape optimization, rather than LC orientation optimization. It uses the same design domain and boundary conditions as the previous example. However, here, the domain contains regions wherein the LCs orientation is either $90^{\circ }$ or $0^{\circ }$, cf. Figure 6, and we seek a bell-curve displacement $u_{Y}^{*}\left(X\right)=20.0/\left(1.0+e^{{\left(X_{1}-L/2\right)}^{2}/20.0)}\right)\cdot e_{2}$ over $\Gamma_{\tau }$. The initial design assigns $h_{1}=1.5$, $h_{2}=2.0$, $l_{1}=5.0$, and $l_{2}=4.5$. In the optimization, the shapes of the material interfaces are optimized; the outer boundary is fixed, i.e., $\Gamma_{D}^{\Delta X}=\partial \Omega$. Additionally, the amount of material of each phase is also fixed. The optimization problem reads:

(24) $$\begin{array}{cc} \min_{\Delta X}\;\;\;\;\;\;\tau & =\int_{\Gamma_{\tau }}{\left|u-u_{Y}^{*}\right|}^{2}d\Gamma \\ \mathrm{such}\;\mathrm{that}\;\;\;g_{1} & =\frac{V_{⊥}}{V_{⊥}^{0}}-1.0\leq 0\\ {\Delta X}_{1},{\Delta X}_{2},\ldots ,{\Delta X}_{2n_{nodes}} & \in [{\Delta X}_{min},{\Delta X}_{max}]. \end{array}$$

The constraint $g_{1}$ ensures that the volume of the material regions with angles at $90^{\circ }$, $V_{⊥}$, remains the same through the optimization, i.e., $V_{⊥}=V_{⊥}^{0}$, where $V_{⊥}^{0}$ is the initial volume of this region. The lower and upper limits are defined as ${\Delta X}_{min}=-2.0$ and ${\Delta X}_{max}=+2.0$.

**Figure 7:**
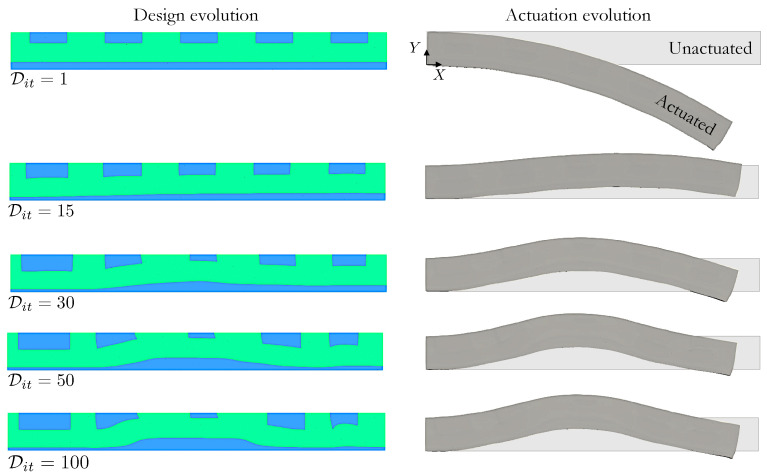
(**Left**) Snapshots of the evolution of leaping structure colored by angle alignment as described in Figure 6; and (**right**) their unactuated and actuated configurations in light gray and dark gray, respectively.

Snapshots of the design at iterates $D_{it}$ on the left side of Figure 7 show noticeable shape variations that favor more LC mesogens oriented at $0.0^{\circ }$ near the center of the bottom edge, i.e., the bottom region thickens in a nonuniform manner. The shapes of the top rectangles change independently to further optimize actuation performance, i.e., they grow near the inflection points of $\Gamma_{\tau }$ to aid in generating the desired deformation. The undeformed and actuated LCE strip configurations for the initial and shape optimized designs shown on the right side of Figure 7 clearly illustrate that controlled shape changes of the material interface can generate considerable actuation response. This example also demonstrates how the optimization can accommodate fabrication constraints, like restricted LC orientation choices, while still leveraging the design freedom offered by 3D printing technologies.

### 4.3. LC Orientation and Shape Optimization for Energy Absorbing Lattice Structures

In this example, we design an LCE lattice structure, optimizing both its LC orientation and shape. Unlike the previous example where the domain geometry Ω was fixed and only the material interfaces were shape optimized, here, the boundary of the lattice morphs concurrently with the orientation angle throughout the optimization.

**Figure 8:**
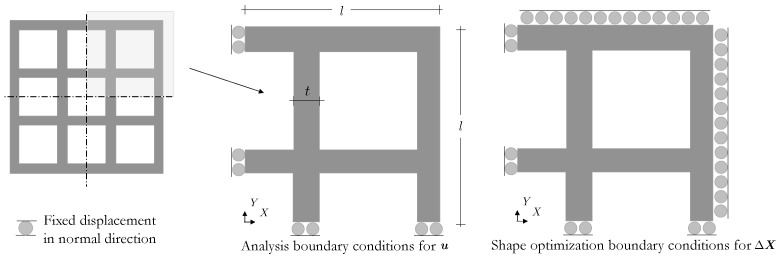
A quarter of the 3×3 LCE lattice design domain (**left**) is simulated. The onsets at the **center** and **right** depict the boundary conditions for the analysis for u and shape perturbation for $\tilde{\Delta X}$.

We consider the two-dimensional representation of the log pile lattice structure illustrated in Figure 8. Only a quarter of the 3×3 cell domain with length l=15 [mm] and thickness t=2.0 [mm] is simulated. Rollers along the left and bottom boundaries are applied to enforce domain symmetry in the analysis; and rollers along all external boundaries are applied to the shape filter (i.e., $\Gamma_{D}^{X}$ in Equation (5)) to limit the shape changes within a confined subregion in the optimization. The LC orientation and shape optimization maximizes the energy absorbed by the lattice in the actuated state while maintaining a constant volume, i.e.,

(25) $$\begin{array}{cc} \max_{\alpha ,\Delta X}\Psi =\min_{\alpha ,\Delta X}-\Psi & =-\int_{\Omega (X)}\psi d\Omega \\ \mathrm{such}\;\mathrm{that}\;\;\;g_{1} & =\frac{V}{V^{0}}-1.0\leq 0\\ \alpha_{1},\alpha_{2},\ldots ,\alpha_{n_{elems}} & \in [\alpha_{min},\alpha_{max}],\\ {\Delta X}_{1},{\Delta X}_{2},\ldots ,{\Delta X}_{2n_{nodes}} & \in [{\Delta X}_{min},{\Delta X}_{max}]. \end{array}$$

The strain energy density ψ is defined in Equation (1), V is the volume of the evolving design, and $V^{0}$ is the volume of the initial design. Both the LC orientation and shape design parameters are filtered as explained above. The lower and upper limits of the shape parameters are ${\Delta X}_{min}=-1.125$, and ${\Delta X}_{max}=+1.125$, respectively.

**Figure 9:**
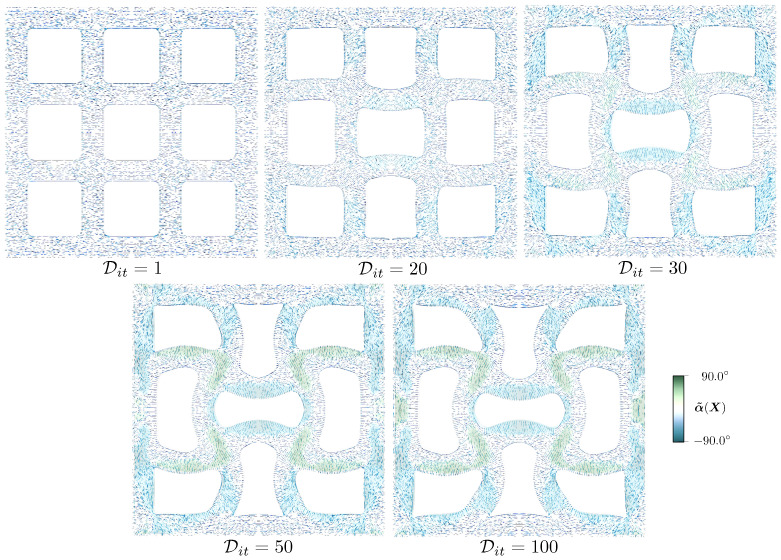
Evolution of lattice structure design. The LC orientation and domain shape are optimized concurrently.

We provide designs of selected optimization iterates $D_{it}$ in Figure 9 to show the concurrent evolution of the LC orientation and domain shape. A relatively uniform connector thickness is preserved despite not being a restriction of the shape parameterization, and curved connectors replace the initially straight ones. The LC orientation varies from its lower to the upper limits across some of the connectors.

**Figure 10:**
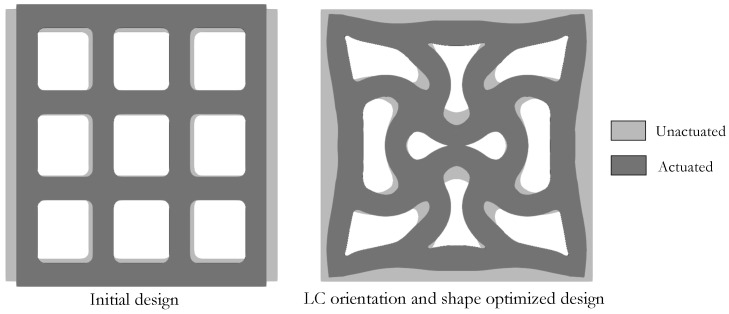
Initial (**left**) and optimized (**right**) lattice structures in the unactuated and actuated states.

Figure 10 displays the initial and optimized structures in their unactuated and actuated states. Note that restricting shape changes to the provided upper and lower limits was necessary to prevent self contact between the connectors. Certainly, this class of applications would benefit from including contact in the analysis as discussed in more detail in Section 5. Nonetheless, this optimization provides an optimal design that can absorb two orders of magnitude more energy (i.e., ${\Psi |}_{D_{it}=100}{=117.1\;\Psi |}_{D_{it}=1}$) compared to the initial design.

### 4.4. Actuation-Driven Compliant Mechanism Design Optimization

**Figure 11:**
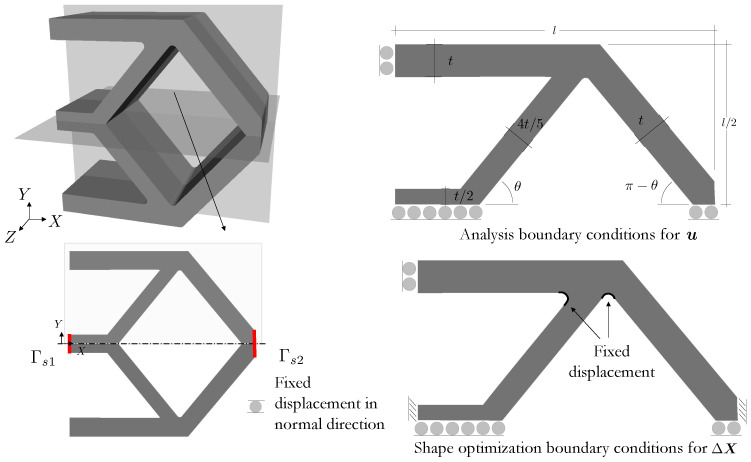
Compliant mechanism design problem: planes of symmetry in 3D domain (**top left**), cross-section (**bottom left**), and onsets detailing the boundary conditions for the analysis for u (**top right**) and shape perturbation for $\tilde{\Delta X}$ (**bottom right**).

Designing compliant mechanisms that invert displacement/force with LCE structures can offer advantages in terms of adaptability, energy absorption, and customization, among others. In this final example, we optimize the LC orientation and shape of the three-dimensional structure depicted in Figure 11. A quarter of the design domain is simulated due to domain symmetry. This geometry is described by l=15.0 [mm], t=1.5 [mm], $\theta =55.0^{\circ }$, and an out-of-plane thickness of 3.0 [mm]. The design problem minimizes the distance between the surfaces $\Gamma_{s1}$ and $\Gamma_{s2}$ (cf. bottom left of Figure 11) while maintaining a constant volume, i.e.,

(26) $$\begin{array}{cc} \min_{\alpha ,\Delta X}\;\;\;\;\;\;\tau & =\int_{\Gamma_{\tau_{s1}}}{\left|u\left(X\right)-u\left(X+le_{1}\right)\right|}^{2}d\Gamma \\ \mathrm{such}\;\mathrm{that}\;\;\;g_{1} & ={\left(\frac{V}{V^{0}}-1.0\right)}^{2}\leq 0\\ \alpha_{1},\alpha_{2},\ldots ,\alpha_{n_{elems}} & \in [\alpha_{min},\alpha_{max}],\\ {\Delta X}_{1},{\Delta X}_{2},\ldots ,{\Delta X}_{3n_{nodes}} & \in [{\Delta X}_{min},{\Delta X}_{max}]. \end{array}$$

The lower and upper limits for the shape changes are ${\Delta X}_{min}=-1.725$, and ${\Delta X}_{max}=1.725$.

**Figure 12:**
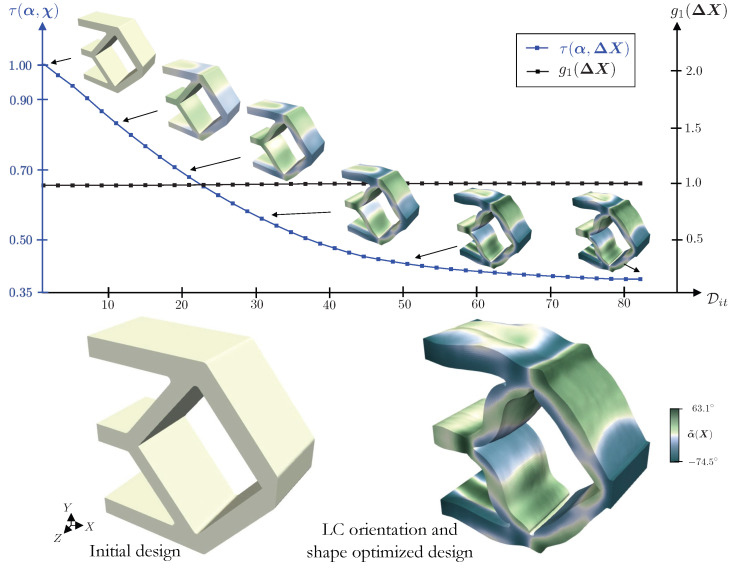
Evolution of the objective and constraint for the compliant mechanism design problem.

A smooth evolution of the design is observed in the plot of Figure 12, which illustrates iterates in their unactuated states and their LC orientations. The initial and optimized designs are presented at the bottom of Figure 12. In the optimized design, the top and bottom legs curve in the *Z*-direction, while the inclined legs near $\Gamma_{s1}$ curve in the XY-plane. In addition, the horizontal leg at the center is tapered inwards. Modest shape changes are observed in the remaining sections. The LC orientation field aids in the actuation by defining adjacent regions with drastically different alignments.

**Figure 13:**
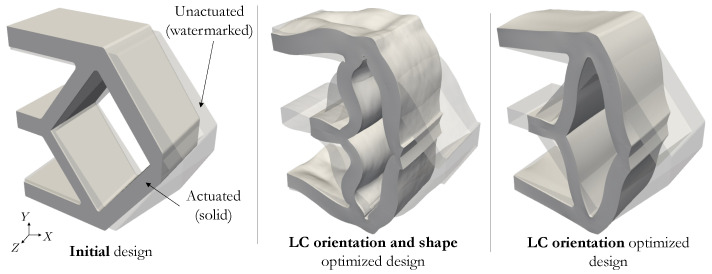
Unactuated and actuated states for initial (**left**), LC orientation and shape optimal (**center**), and LC orientation only optimal (**right**) designs.

The actuated design configurations of Figure 13 clearly demonstrate the enhanced performance of the LC orientation and shape optimized design. Note that this is also the case when compared to an LC orientation-only optimized design, i.e., without shape optimization. The normalized distance measure of the LC orientation-only design is τ=0.486; it reduces to τ=0.381 for the combined LC orientation and shape design. This final example underscores the superior performance by expanding the design space to include both LC orientation and shape (∼22% reduction in distance between the surfaces $\Gamma_{s1}$ and $\Gamma_{s2}$) instead of only optimizing the LC orientation (∼10% reduction). Furthermore, it highlights our optimization framework’s capacity to effectively solve such problems.

## 5. Discussion and Future Work

The gradient-based optimization for active LCE (and similar active material) structures is a multidisciplinary challenge. Indeed, the case studies presented above cover a range of representative applications but represent only a fraction of them. To effectively solve computational design optimization problems with more complex geometries, more pronounced actuations, and more intricate optimization formulations, the following should be considered regarding the material model, simulation, sensitivity analysis, and optimization methodology.

- The constitutive model selection can be a difficult task for the simulation of LCEs, and responsive materials in general. Advanced applications such as controlled locomotion [1,3] require intricate material models (e.g., mutiscale modeling [52,62,63]) to accurately predict the LCE motion. These may require heat transfer simulations to design thermally actuated LCEs, for example. Furthermore, given the diverse stimuli that LCEs respond to, the constitutive model employed must accurately capture their multi-stimuli response in optimization scenarios where designs are influenced by more than one stimulus. Other physical phenomena such as rate effects may also need to be considered, e.g., high strain rate compression/impact. Fortunately, emerging AI-based trends can ease some of this burden. For example, machine learning can help characterize LCE materials and provide more accurate constitutive models [64]. Progress has been made in physics-informed neural networks (PINNs) for hyperelastic materials [65,66], and strategies to generate models that obey the laws of physics are being explored under the umbrella of constitutive artificial neural networks (CANNs, [67,68]). Once open questions regarding data quality and diversity for multi-physics responses are resolved, future developments could integrate classical physics-based methods with the mentioned emerging machine learning methods to more accurately simulate these complex materials.
- Simulation challenges associated with the robust numerical analysis of complex geometries, nontrivial time-dependent boundary and interface conditions, including contact, and parameterized material properties and shapes, need to be addressed. Appropriate linear and nonlinear solvers, and preconditioning strategies must be selected as well as stabilization terms added to avoid undesirable pathologies, e.g., spurious oscillations, and locking [69,70]. Existing literature directed towards this issue for LCEs or similar responsive structures is lacking, except in the context of simplified benchmarks [52].
- Implementing adjoint sensitivity analysis that can accommodate the dynamic nature of advanced systems (e.g., controlled actuation in time of an LCE structure) introduces another challenge, notably efficient check-pointing schemes [71]. Three-dimensional design applications characterized by extensive arrays of design parameters call for approaches that transcend rudimentary high-level programming or reliance on commercial software [72]. Implementations must use alternative HPC simulation and design libraries and AD tools to solve more complex engineering problems.
- Alternative shape optimization methodologies using the level set method (LSM, [73,74,75]) allow for topological changes and thus, potentially generate better performing designs. The increased design freedom of LSMs is also favorable for designing systems for which a suitable initial design is unknown, or organic geometries are expected (e.g., biomimicry engineering). For LCE structures, the more flexible parameterization offered by LSMs can accommodate additional manufacturing constraints such as aligning the LC fibers with the printing direction. However, considerable additional effort both in the analysis and optimization is needed to guarantee a robust framework [76,77,78].

## 6. Conclusions

We propose a gradient-based framework to optimize the liquid crystal orientation and shape of liquid crystal elastomer structures. To simulate the structure motion when it is exposed to external stimuli, we combined nonlinear kinematics material and models with a quasi static finite element analysis. A well-posed optimization problem is formulated by using an energy filter to eliminate highly oscillatory design fields. We presented the necessary sensitivity analyses and designed two- and three-dimensional structures to achieve the desired target shape changes and maximal strain energy. Optimal designs performing between one to two orders of magnitude better than the initial designs were obtained when using combined liquid crystal orientation and shape optimization. Finally, we have provided a discussion summarizing the challenges encountered, as well as considerations for advanced applications and future work.

## Figures and Tables

**Table 1:** Material properties and other parameters for LCE analysis.

Parameter	Value
Nematic–isotropic coupling parameter, β	$5.75\times 10^{5}$ [N/m^2^]
Elastic modulus, E	$9.34\times 10^{6}$ [N/m^2^]
Poisson’s ratio, ν	0.48
Initial order parameter, $S^{0}$	0.40
Total actuation time, $t_{max}$	1.0
Newton tolerance, $\epsilon_{R}$	$1.0\times 10^{-6}$

## Data Availability

No new data were created or analyzed in this study. Data sharing is not applicable to this article.

## References

[1] Ford M.J., Porcincula D.H., Telles R., Mancini J.A., Wang Y., Rizvi M.H., Loeb C.K., Moran B.D., Tracy J.B., Lewis J.A. (2024). Movement with light: Photoresponsive shape morphing of printed liquid crystal elastomers. Matter.

[2] Yuan C., Roach D.J., Dunn C.K., Mu Q., Kuang X., Yakacki C.M., Wang T., Yu K., Qi H.J. (2017). 3D printed reversible shape changing soft actuators assisted by liquid crystal elastomers. Soft Matter.

[3] Hebner T.S., Korner K., Bowman C.N., Bhattacharya K., White T.J. (2023). Leaping liquid crystal elastomers. Sci. Adv..

[4] Konya A., Gimenez-Pinto V., Selinger R.L. (2016). Modeling defects, shape evolution, and programmed auto-origami in liquid crystal elastomers. Front. Mater..

[5] Sydney Gladman A., Matsumoto E.A., Nuzzo R.G., Mahadevan L., Lewis J.A. (2016). Biomimetic 4D printing. Nat. Mater..

[6] George D., Madou M.J., Hernandez E.A.P. (2020). Programmable self-foldable films for origami-based manufacturing. Smart Mater. Struct..

[7] Kotikian A., Morales J.M., Lu A., Mueller J., Davidson Z.S., Boley J.W., Lewis J.A. (2021). Innervated, self-sensing liquid crystal elastomer actuators with closed loop control. Adv. Mater..

[8] Doi H., Takahashi K.Z., Tagashira K., Fukuda J.i., Aoyagi T. (2019). Machine learning-aided analysis for complex local structure of liquid crystal polymers. Sci. Rep..

[9] Hu Q., Chen K., Liu F., Zhao M., Liang F., Xue D. (2022). Smart Materials Prediction: Applying Machine Learning to Lithium Solid-State Electrolyte. Materials.

[10] Ortigosa R., Martínez-Frutos J., Gil A.J. (2023). Programming shape-morphing electroactive polymers through multi-material topology optimisation. Appl. Math. Model..

[11] Bhattacharyya A., Kim J., Alacoque L.R., James K.A. (2024). Design Synthesis of a 4D-Printed Self-Tying Knot with Programmable Morphology. J. Mech. Des..

[12] Kirchdoerfer T., Ortiz M. (2016). Data-driven computational mechanics. Comput. Methods Appl. Mech. Eng..

[13] Kumar S., Kochmann D.M. (2022). What machine learning can do for computational solid mechanics. Current Trends and Open Problems in Computational Mechanics.

[14] Brodnik N., Muir C., Tulshibagwale N., Rossin J., Echlin M., Hamel C., Kramer S., Pollock T., Kiser J., Smith C. (2023). Perspective: Machine learning in experimental solid mechanics. J. Mech. Phys. Solids.

[15] Kang Z., James K.A. (2022). Multiphysics design of programmable shape-memory alloy-based smart structures via topology optimization. Struct. Multidiscip. Optim..

[16] Geiss M.J., Boddeti N., Weeger O., Maute K., Dunn M.L. (2019). Combined Level-Set-XFEM-Density Topology Optimization of Four-Dimensional Printed Structures Undergoing Large Deformation. J. Mech. Des..

[17] Ortigosa R., Martínez-Frutos J. (2021). Topology optimisation of stiffeners layout for shape-morphing of dielectric elastomers. Struct. Multidiscip. Optim..

[18] Fuchi K., Ware T.H., Buskohl P.R., Reich G.W., Vaia R.A., White T.J., Joo J.J. (2015). Topology optimization for the design of folding liquid crystal elastomer actuators. Soft Matter.

[19] Yan J., Xiang R., Kamensky D., Tolley M.T., Hwang J.T. (2022). Topology optimization with automated derivative computation for multidisciplinary design problems. Struct. Multidiscip. Optim..

[20] Koda M., Dogru A.H., Seinfeld J.H. (1979). Sensitivity analysis of partial differential equations with application to reaction and diffusion processes. J. Comput. Phys..

[21] Tortorelli D.A., Michaleris P. (1994). Design sensitivity analysis: Overview and review. Inverse Probl. Eng..

[22] Van Keulen F., Haftka R., Kim N.H. (2005). Review of options for structural design sensitivity analysis. Part 1: Linear systems. Comput. Methods Appl. Mech. Eng..

[23] Allaire G. (2015). A review of adjoint methods for sensitivity analysis, uncertainty quantification and optimization in numerical codes. Ing. L’Automob..

[24] Sienz J., Hinton E. (1997). Reliable structural optimization with error estimation, adaptivity and robust sensitivity analysis. Comput. Struct..

[25] vom Lehn F., Cai L., Pitsch H. (2019). Sensitivity analysis, uncertainty quantification, and optimization for thermochemical properties in chemical kinetic combustion models. Proc. Combust. Inst..

[26] Borgonovo E., Plischke E. (2016). Sensitivity analysis: A review of recent advances. Eur. J. Oper. Res..

[27] Barrera Cruz J.L., Maute K.K. Immersed Boundary Analysis of Models with Internal State Variables: Applications to Hydrogels. Proceedings of the ECCOMAS Congress 2022—8th European Congress on Computational Methods in Applied Sciences and Engineering.

[28] Chung S.H., Fourment L., Chenot J.L., Hwang S. (2003). Adjoint state method for shape sensitivity analysis in non-steady forming applications. Int. J. Numer. Methods Eng..

[29] Wang W., Clausen P.M., Bletzinger K.U. (2017). Efficient adjoint sensitivity analysis of isotropic hardening elastoplasticity via load steps reduction approximation. Comput. Methods Appl. Mech. Eng..

[30] Ozaki I., Kimura F., Berz M. (1995). Higher-order sensitivity analysis of finite element method by automatic differentiation. Comput. Mech..

[31] Hou G., Satyanarayana A., Tiwari S. First-and second-order sensitivity analysis of finite element equations via automatic differentiation. Proceedings of the 7th AIAA/USAF/NASA/ISSMO Symposium on Multidisciplinary Analysis and Optimization.

[32] Rathgeber F., Ham D.A., Mitchell L., Lange M., Luporini F., McRae A.T., Bercea G.T., Markall G.R., Kelly P.H. (2016). Firedrake: Automating the finite element method by composing abstractions. ACM Trans. Math. Softw. (Toms).

[33] Arndt D., Bangerth W., Davydov D., Heister T., Heltai L., Kronbichler M., Maier M., Pelteret J.P., Turcksin B., Wells D. (2021). The deal. II finite element library: Design, features, and insights. Comput. Math. Appl..

[34] Anderson R., Andrej J., Barker A., Bramwell J., Camier J.S., Cerveny J., Dobrev V., Dudouit Y., Fisher A., Kolev T. (2021). MFEM: A modular finite element methods library. Comput. Math. Appl..

[35] Bramwell J., White C., Essman J., Mish S., Chapman A., Talamini B., Wong J., Chin E., Tupek M. (2023). Serac. https://github.com/LLNL/serac/.

[36] Brighenti R., McMahan C.G., Cosma M.P., Kotikian A., Lewis J.A., Daraio C. (2021). A micromechanical-based model of stimulus responsive liquid crystal elastomers. Int. J. Solids Struct..

[37] Mihai L.A., Goriely A. (2021). Instabilities in liquid crystal elastomers. Mrs Bull..

[38] Bartels S., Griehl M., Keck J., Neukamm S. (2022). Modeling and simulation of nematic LCE rods. arXiv.

[39] Lee V., Wihardja A., Bhattacharya K. (2023). A macroscopic constitutive relation for isotropic-genesis, polydomain liquid crystal elastomers. J. Mech. Phys. Solids.

[40] Finkelmann H., Greve A., Warner M. (2001). The elastic anisotropy of nematic elastomers. Eur. Phys. J..

[41] Mbanga B.L., Ye F., Selinger J.V., Selinger R.L. (2010). Modeling elastic instabilities in nematic elastomers. Phys. Rev..

[42] Wang Z., Chehade A.E.H., Govindjee S., Nguyen T.D. (2022). A nonlinear viscoelasticity theory for nematic liquid crystal elastomers. J. Mech. Phys. Solids.

[43] Duffy D., McCracken J.M., Hebner T.S., White T.J., Biggins J.S. (2023). Lifting, loading, and buckling in conical shells. Phys. Rev. Lett..

[44] Cosma M.P., Brighenti R. (2022). Controlled morphing of architected liquid crystal elastomer elements: Modeling and simulations. Mech. Res. Commun..

[45] Mihai L.A. (2022). Liquid Crystal Elastomers. Stochastic Elasticity: A Nondeterministic Approach to the Nonlinear Field Theory.

[46] Bartels S., Griehl M., Neukamm S., Padilla-Garza D., Palus C. (2023). A nonlinear bending theory for nematic LCE plates. Math. Model. Methods Appl. Sci..

[47] Li W., Zhang X.S. (2023). Arbitrary curvature programming of thermo-active liquid crystal elastomer via topology optimization. Comput. Methods Appl. Mech. Eng..

[48] Li S., Librandi G., Yao Y., Richard A.J., Schneider-Yamamura A., Aizenberg J., Bertoldi K. (2021). Controlling liquid crystal orientations for programmable anisotropic transformations in cellular microstructures. Adv. Mater..

[49] Zhu W., Shelley M., Palffy-Muhoray P. (2011). Modeling and simulation of liquid-crystal elastomers. Phys. Rev..

[50] Luo C., Calderer M.C. (2009). Numerical Study of Liquid Crystal Elastomer Using Mixed Finite Element Method. arXiv.

[51] Nochetto R.H., Walker S.W., Zhang W. (2017). A finite element method for nematic liquid crystals with variable degree of orientation. Siam J. Numer. Anal..

[52] Soltani M., Raahemifar K., Nokhosteen A., Kashkooli F.M., Zoudani E.L. (2021). Numerical methods in studies of liquid crystal elastomers. Polymers.

[53] Park S., Oh Y., Moon J., Chung H. (2023). Recent Trends in Continuum Modeling of Liquid Crystal Networks: A Mini-Review. Polymers.

[54] Holzapfel G.A. (2002). Nonlinear Solid Mechanics: A Continuum Approach for Engineering Science.

[55] Swartz K.E., Mittal K., Schmidt M., Barrera J.L., Watts S., Tortorelli D.A. (2023). Yet another parameter-free shape optimization method. Struct. Multidiscip. Optim..

[56] Sigmund O., Maute K. (2013). Topology optimization approaches. Struct. Multidiscip. Optim..

[57] Lazarov B.S., Sigmund O. (2011). Filters in topology optimization based on Helmholtz-type differential equations. Int. J. Numer. Methods Eng..

[58] Smith D.E., Tortorelli D.A., Tucker III C.L. (1998). Optimal design for polymer extrusion. Part I: Sensitivity analysis for nonlinear steady-state systems. Comput. Methods Appl. Mech. Eng..

[59] Ghysels P., Synk R. (2022). High performance sparse multifrontal solvers on modern GPUs. Parallel Comput..

[60] Svanberg K. (1987). The method of moving asymptotes—a new method for structural optimization. Int. J. Numer. Methods Eng..

[61] LLNL (2023). The Livermore Design Optimization (LiDO) Code. https://str.llnl.gov/2018-03.

[62] Moon J., Shin H., Baek K., Choi J., Cho M. (2018). Multiscale modeling of photomechanical behavior of photo-responsive nanocomposite with carbon nanotubes. Compos. Sci. Technol..

[63] Kalina K.A., Ra<i>β</i>loff A., Wollner M., Metsch P., Brummund J., Kästner M. (2023). Multiscale modeling and simulation of magneto-active elastomers based on experimental data. Phys. Sci. Rev..

[64] Kovachki N., Liu B., Sun X., Zhou H., Bhattacharya K., Ortiz M., Stuart A. (2022). Multiscale modeling of materials: Computing, data science, uncertainty and goal-oriented optimization. Mech. Mater..

[65] Abueidda D.W., Koric S., Guleryuz E., Sobh N.A. (2023). Enhanced physics-informed neural networks for hyperelasticity. Int. J. Numer. Methods Eng..

[66] Linden L., Klein D.K., Kalina K.A., Brummund J., Weeger O., Kästner M. (2023). Neural networks meet hyperelasticity: A guide to enforcing physics. J. Mech. Phys. Solids.

[67] Linka K., Kuhl E. (2023). A new family of Constitutive Artificial Neural Networks towards automated model discovery. Comput. Methods Appl. Mech. Eng..

[68] Tac V., Linka K., Sahli-Costabal F., Kuhl E., Tepole A.B. (2023). Benchmarks for physics-informed data-driven hyperelasticity. arXiv.

[69] Hughes T.J., Mallet M., Akira M. (1986). A new finite element formulation for computational fluid dynamics: II. Beyond SUPG. Comput. Methods Appl. Mech. Eng..

[70] Franca L.P., Do Carmo E.G.D. (1989). The Galerkin gradient least-squares method. Comput. Methods Appl. Mech. Eng..

[71] Griewank A., Walther A. (2000). Algorithm 799: Revolve: An implementation of checkpointing for the reverse or adjoint mode of computational differentiation. ACM Trans. Math. Softw. (Toms).

[72] Pyzer-Knapp E.O., Pitera J.W., Staar P.W., Takeda S., Laino T., Sanders D.P., Sexton J., Smith J.R., Curioni A. (2022). Accelerating materials discovery using artificial intelligence, high performance computing and robotics. NPJ Comput. Mater..

[73] Duysinx P., Van Miegroet L., Jacobs T., Fleury C. (2006). Generalized shape optimization using X-FEM and level set methods. Proceedings of the IUTAM Symposium on Topological Design Optimization of Structures, Machines and Materials: Status and Perspectives.

[74] Barrera J.L., Geiss M.J., Maute K. (2020). Hole seeding in level set topology optimization via density fields. Struct. Multidiscip. Optim..

[75] Schmidt M., Barrera J.L., Swartz K., Mittal K., Tortorelli D. (2024). Level-set topology optimization with PDE-generated conformal meshes. Struct. Multidiscip. Optim..

[76] Geiss M.J., Barrera J.L., Boddeti N., Maute K. (2019). A regularization scheme for explicit level-set XFEM topology optimization. Front. Mech. Eng..

[77] Soghrati S., Barrera J.L. (2016). On the application of higher-order elements in the hierarchical interface-enriched finite element method. Int. J. Numer. Methods Eng..

[78] Barrera J.L., Maute K. (2020). Ambiguous phase assignment of discretized 3D geometries in topology optimization. Comput. Methods Appl. Mech. Eng..

